# Time-structured detection of pre-event cardiovascular instability using CPAP-derived Cheyne–Stokes breathing

**DOI:** 10.1093/ehjdh/ztag108

**Published:** 2026-07-02

**Authors:** Kimimasa Saito, Yoko Takamatsu

**Affiliations:** Saito Naika Kokyukika, Mie Sleep Clinic, 446 Sogo, Obata-cho, Ise-shi, Mie 519-0502, Japan; Saito Naika Kokyukika, Mie Sleep Clinic, 446 Sogo, Obata-cho, Ise-shi, Mie 519-0502, Japan

**Keywords:** Cheyne–Stokes breathing, CPAP telemonitoring, Cardiovascular instability, Heart failure, Digital biomarker, Time-structured detection

## Abstract

**Aims:**

Continuous positive airway pressure (CPAP) telemonitoring provides daily respiratory metrics, including Cheyne–Stokes breathing percentage (CSB%), which may reflect ventilatory–circulatory instability. However, inter-individual variability limits the clinical applicability of fixed thresholds. We developed a time-structured detection architecture using CPAP telemonitoring time-series data and evaluated its ability to identify temporal patterns preceding cardiovascular and cerebrovascular events.

**Methods and results:**

In this retrospective observational study, 1265 patients with obstructive sleep apnoea undergoing CPAP telemonitoring were analysed. Daily CSB% values were smoothed using a 3-day moving average and evaluated relative to individualized dynamic baselines. The detection framework consisted of two complementary components: identification of sustained deviation from baseline and detection of abrupt pre-event surges. Central apnoea predominance, assessed using the central apnoea index, was incorporated as a hierarchical escalation layer. Among 25 adjudicated cardiovascular and cerebrovascular events in 20 patients (heart failure 11, atrial fibrillation 7, cerebrovascular accident 7), the architecture detected 23 events (92.0%) within the predefined D−28 window. Median lead time was 12.0 days (IQR 2.8–24.0). False-positive alerts were concentrated within a subset of individuals. Hierarchical filtering reduced alerts by 94.9% relative to the baseline signal layer while preserving event enrichment. Distinct temporal phenotypes, including trajectory-dominant and spike-dominant patterns, were observed across disease categories, consistent with disease-specific pre-event dynamics.

**Conclusion:**

CPAP-derived CSB% may function as a time-structured digital biomarker reflecting evolving ventilatory–circulatory instability. A trajectory-based detection architecture may enable early identification of cardiovascular instability while maintaining operational feasibility in large-scale telemonitoring environments.

## Graphical Abstract

**Graphical Abstract:**
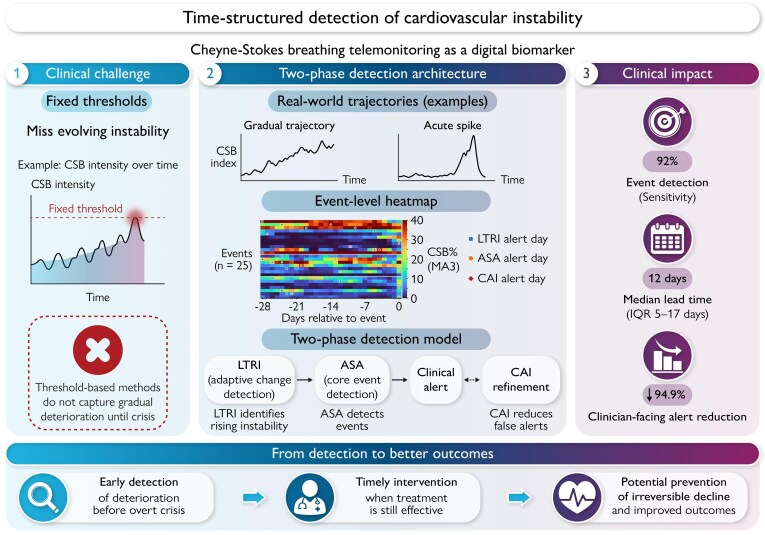

## Introduction

Continuous positive airway pressure (CPAP) telemonitoring provides daily respiratory metrics, including Cheyne–Stokes breathing (CSB), which has been associated with cardiovascular risk.^1–3^ However, translation of these findings into clinical practice remains limited due to inter-individual variability and reliance on static threshold-based interpretation.

Fixed-threshold approaches are simple but often fail to capture the dynamic nature of cardiopulmonary instability, particularly in chronic cardiovascular conditions where haemodynamic deterioration has been shown to evolve gradually prior to decompensation.^4^ Continuous monitoring enables detection of temporal patterns such as drift, fluctuation, and acceleration, which may provide earlier signals.

Cheyne–Stokes breathing reflects ventilatory–circulatory instability arising from interactions between respiratory control, cardiac function, and circulatory delay.^5,6^ Cardiovascular deterioration often develops over days to weeks before overt clinical events,^4^ suggesting static metrics may not capture this subclinical phase. Accordingly, temporal dynamics may provide more relevant information.

CPAP-derived CSR/CSB is associated with heart failure and adverse outcomes,^7^ consistent with prior studies.^8,9^

We therefore hypothesized that CSB trajectories, rather than absolute values, may indicate impending cardiovascular and cerebrovascular events. In this study, we evaluated event-level detection within a predefined D−28 window, characterized time-structured phenotypes across cardiovascular events, and assessed operational feasibility in a large CPAP telemonitoring cohort (UMIN000058672).

## Methods

This retrospective observational study included patients with obstructive sleep apnoea (OSA) undergoing CPAP telemonitoring at our institution between 19 August 2024 and 31 December 2025. CPAP therapy was delivered using the DreamStation Auto BiPAP device (Philips Respironics), with data synchronized via eHomeCare and analyzed using Care Orchestrator.

A total of 1265 patients were included, among whom 25 adjudicated cardio-cerebrovascular events in 20 patients [heart failure (HF) 11, atrial fibrillation (AF) 7, cerebrovascular accident (CVA) 7] were analyzed. Events were defined as hospital admission or outpatient intervention for HF, AF, or CVA. Continuous deteriorations without recovery were aggregated as a single event, whereas recurrent AF following sinus rhythm restoration was treated as distinct. Analyses were conducted at the event level, although residual within-subject dependence cannot be fully excluded. The index date (D0) was defined as three days prior to admission or intervention. HF events were classified as *de novo* or chronic decompensation.

Daily telemonitoring variables included Cheyne–Stokes breathing percentage (CSB%), 3-day moving average of CSB% (MA3-CSB), central apnoea index (CAI), 3-day moving average of CAI (CAI-MA3), and apnoea–hypopnea index (AHI). Moving averages were used to reduce short-term variability while preserving trajectory dynamics. Apnoea subtype classification was based on device-derived algorithms.

To mitigate inter-individual variability and seasonal fluctuation, individualized dynamic baselines were defined. For event cases, baseline was calculated as the median MA3-CSB during days −60 to −30 relative to D0; for non-event cases, it was defined during days 31–60 after telemonitoring initiation to ensure temporal independence and avoid post-event contamination. Patients were stratified into four vulnerability bands: S (<1.5%), A (1.5–<5%), B (5–<10%), and C (≥10%).

A two-phase temporal alert architecture was implemented using daily rolling evaluation. LTRI required MA3-CSB ≥2%, three consecutive increases, and band-specific absolute and relative increases from baseline. Thresholds were defined *a priori* based on physiological plausibility and exploratory signal characterization rather than formal optimization procedures. Band S was excluded from LTRI evaluation. LTRI lead time was defined as the interval from first activation to D0.

ASA detected abrupt pre-event surges using a 7-day rolling median and required MA3-CSB ≥3%, relative elevation above the median threshold, and three-day persistence. ASA lead time was defined analogously.

Central apnoea predominance (MA3-CAI ≥5 and CAI/AHI ≥0.5 for ≥2 days) was used as a clinician-level escalation factor. Event trajectories were classified as *de novo* spike, chronic trajectory, mixed, or central apnoea predominance phenotypes.

Notifications were hierarchically structured. Initial LTRI triggered a single automated notification with a 20-day cooldown, whereas clinician-level alerts required LTRI and/or ASA combined with central apnoea predominance.

False-positive alerts were defined outside the D−28 to D0 window. LTRI evaluation was restricted to Bands A–C with ≥3 consecutive days, whereas ASA required a valid 7-day median window. Alerts within 7 days were clustered as a single episode, and both day-level and episode-level false-positive rates were calculated.

Operational burden was assessed by estimating daily alert-generating patients and clinician-level notifications, with projections scaled to a 1300-patient cohort.

## Results

Among 25 adjudicated cardiovascular and cerebrovascular events in 20 patients (HF 11, AF 7, CVA 7), the two-phase alert architecture achieved an overall detection rate of 92.0% within the D−28 window. Detection rates were 90.9% for HF, 85.7% for AF, and 100.0% for CVA. The median lead time for two-phase detection was 12.0 days (IQR 2.8–24.0). When clinician-level notification criteria were applied, sensitivity decreased to 72.0%, with a median lead time of 6.5 days (IQR 0.0–18.0).

**Figure 1:**
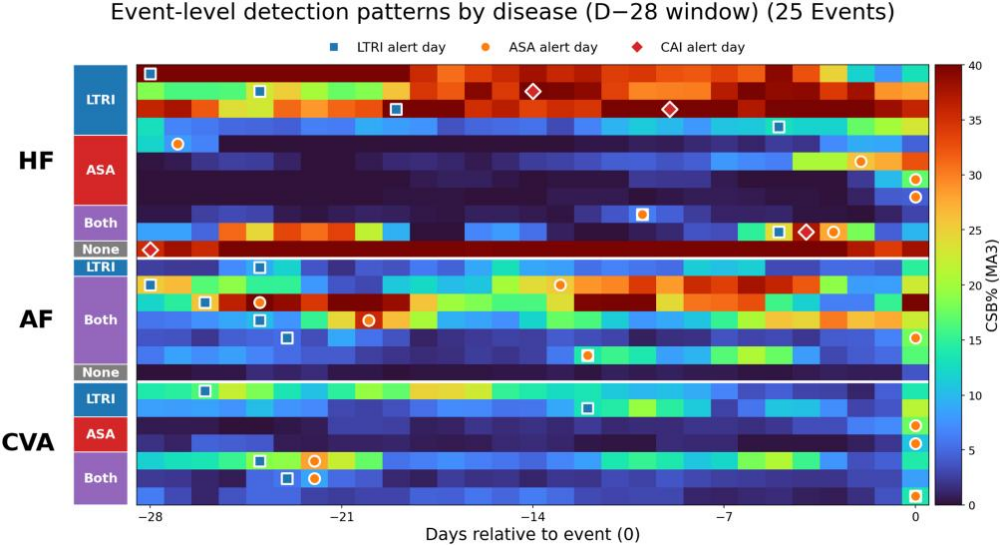
Event-level heatmap of time-aligned trajectories of Cheyne–Stokes breathing preceding cardiovascular events. The figure consists of two aligned panels. The left panel shows a bar representation summarizing event classification or grouping, while the right panel displays a heatmap of temporal trajectories. Each row corresponds to a single adjudicated cardiovascular or cerebrovascular event (*n* = 25), and rows are aligned across panels to represent the same event. The horizontal axis indicates time from D−28 to D0. D0 is defined as three days prior to hospital admission or outpatient intervention to simulate a clinically actionable prediction horizon and to avoid post-event contamination. The heatmap colour scale represents the 3-day moving average of Cheyne–Stokes breathing percentage (MA3-CSB), reflecting the burden of periodic breathing over time. Markers indicate the first detection day according to the time-structured detection framework. Distinct temporal patterns are observed across events, including gradual trajectory-dominant increases and abrupt spike-dominant changes preceding clinical deterioration. The visualization highlights that cardiopulmonary instability evolves through heterogeneous but structured temporal dynamics rather than uniform threshold-based behaviour.

*Figure 1* shows an event-level temporal alignment heatmap centred on D0, displaying the first activation timing of each alert layer—Long-Term Risk Index (LTRI), Acute Spike Alert (ASA), and central apnoea predominance (CAI). Pre-event trajectories were structured across disease categories. AF and CVA were predominantly characterized by progressive LTRI-driven patterns, whereas HF showed heterogeneity, including trajectory-dominant, spike-dominant, and mixed phenotypes.

All *de novo* HF events were detected by ASA, either alone or with LTRI, indicating spike-type dynamics in new-onset HF. In contrast, chronic HF cases more often showed sustained pre-event deviation captured by LTRI. Central apnoea predominance was observed exclusively in HF (4/11), supporting disease-specific ventilatory destabilization.

False-positive analysis showed differences between alert layers. At the day level, the false-positive rate was 6.40% for LTRI (4817/75 259 days) and 0.51% for ASA (2701/526 864 days). After 7-day clustering, these rates decreased to 3.06% and 0.34%, respectively, although LTRI remained more frequent.

Pareto analysis showed that ∼80% of LTRI false-positive days were concentrated in a subset of patients. In operational simulation, the two-phase system generated alerts in 5.66 patients/day in a projected 1300-patient cohort; hierarchical filtering resulted in a 94.9% reduction relative to the baseline signal layer, supporting practical feasibility.

## Discussion

This study demonstrates the feasibility of a time-anchored, two-phase CSB% telemonitoring framework that distinguishes sustained cardiopulmonary deterioration from acute pre-event instability. By separating gradual deviation from individualized baselines (LTRI) and abrupt short-term surges (ASA), the system captures temporal phenotypes preceding cardiovascular events, as visualized in *Figure 1*.

The event-aligned heatmap highlights that pre-event trajectories are structured and disease-specific. Progressive LTRI-dominant patterns were more prominent in AF and CVA, whereas HF exhibited heterogeneous dynamics, including trajectory- and spike-dominant patterns. All *de novo* HF cases were detected by ASA, suggesting abrupt ventilatory destabilization as a feature of new-onset HF. These findings support distinct pre-event temporal patterns rather than a single trajectory.

From a physiological perspective, CSB reflects instability in ventilatory control linked to loop gain and feedback dynamics,^5–9^ potentially preceding clinical deterioration. Unlike downstream metrics such as hypoxic burden, CSB% provides upstream insight into ventilatory–circulatory regulation. The observed lead time is consistent with prior observations that cardiopulmonary or haemodynamic deterioration evolves over days to weeks before clinical events,^4,8^ and heterogeneous temporal phenotypes reflect distinct pathophysiological processes.

Our findings are consistent with prior studies and systematic evidence linking CPAP-derived periodic breathing to cardiovascular risk,^1–3,7–9^ and extend these observations by introducing a time-structured framework. Progressive patterns observed in AF may relate to atrial remodelling processes described previously.^10^

This study reframes CPAP-derived CSB% from a descriptive respiratory metric into a time-structured digital biomarker. The use of a fixed pre-event reference (D0) and restriction to pre-event data enhances interpretability and preserves clinically meaningful lead time. Integration of central apnoea predominance as a post-detection escalation criterion further enriches alerts without increasing notification burden.

Operationally, the hierarchical alert design reduced clinician workload while maintaining detection performance, supporting scalability in routine telemonitoring settings. The concentration of false positives within a subset of patients suggests recurrent temporal variability may carry clinical significance and could support targeted monitoring.

Given the limited number of events, trajectory-based descriptive analysis was used to avoid overfitting, preserving interpretability in a small-event setting. It should therefore be interpreted as a proof-of-concept for a time-structured detection framework rather than a validation study. Generalizability across CPAP platforms requires prospective refinement, and prospective studies are warranted.

## Limitations

This study has several limitations. First, the number of events was limited, restricting statistical power and precluding formal model validation; thus, the findings should be interpreted as hypothesis-generating. Second, the alert architecture and thresholds were predefined based on physiological plausibility and empirical observation rather than formal optimization, and require prospective refinement. Third, the retrospective single-centre design may limit generalizability, and the use of a single CPAP platform raises concerns regarding cross-device applicability. Fourth, apnoea classification relied on device-derived algorithms rather than polysomnography, introducing potential misclassification. Finally, although analyses were primarily descriptive and event-based to avoid overfitting, repeated events within individuals may still introduce residual dependence. Prospective, multicentre validation is warranted.

## Conclusion

This study demonstrates that CPAP-derived CSB% can function as a time-structured digital biomarker, enabling early detection of cardiovascular instability through trajectory-based analysis. The two-phase alert architecture provides clinically interpretable lead time while maintaining low operational burden, supporting integration into routine telemonitoring and warranting prospective validation across diverse clinical settings.

## Data Availability

The data underlying this article cannot be shared publicly because they contain potentially identifiable patient information. De-identified data may be made available from the corresponding author upon reasonable request and with appropriate institutional approval.
